# Stimuli-Responsive Thermally Activated Delayed Fluorescence in Polymer Nanoparticles and Thin Films: Applications in Chemical Sensing and Imaging

**DOI:** 10.3389/fchem.2020.00229

**Published:** 2020-04-09

**Authors:** Nathan R. Paisley, Christopher M. Tonge, Zachary M. Hudson

**Affiliations:** Department of Chemistry, The University of British Columbia, Vancouver, BC, Canada

**Keywords:** TADF, imaging, oxygen sensing, polymer films, polymer nanostructure, temperature sensing

## Abstract

Though molecules exhibiting thermally activated delayed fluorescence (TADF) have seen extensive development in organic light-emitting diodes, their incorporation into polymer nanomaterials and thin films has led to a range of applications in sensing and imaging probes. Triplet quenching can be used to probe oxygen concentration, and the reverse intersystem crossing mechanism which gives rise to TADF can also be used to measure temperature. Moreover, the long emission lifetimes of TADF materials allows for noise reduction in time-gated microscopy, making these compounds ideal for time-resolved fluorescence imaging (TRFI). A polymer matrix enables control over energy transfer between molecules, and can be used to modulate TADF behavior, solubility, biocompatibility, or desirable mechanical properties. Additionally, a polymer's oxygen permeability can be tuned to suit imaging applications in a range of media. Here we review the applications of polymer nanoparticles and films exhibiting TADF in sensing and imaging, demonstrating that this class of materials has great potential beyond electroluminescent devices still waiting to be explored.

## Introduction

Since the demonstration of their utility in organic light-emitting diodes (OLEDs) in 2011 (Endo et al., 2011), molecules exhibiting thermally activated delayed fluorescence (TADF) have generated tremendous research interest (Wu et al., 2009; Goushi et al., 2012; Zhang et al., 2012; Cho et al., 2015; Li et al., 2017; Steinegger et al., 2017; Wong and Zysman-Colman, 2017; Yang et al., 2017; Chen et al., 2018). When used as emitters in OLEDs, internal quantum efficiencies up to 100% can be achieved due to their ability to harvest both singlet and triplet excitons (Jankus et al., 2014; Wu et al., 2016). Concurrently, the incorporation of TADF materials into polymer nanostructures led to a range of emerging applications as sensors and imaging probes (Kochmann et al., 2013; Xiong et al., 2014; Gan et al., 2017; Li et al., 2017; Steinegger et al., 2017; Zhu et al., 2018; Tsuchiya et al., 2019; Tonge et al., 2020). TADF emitters can be incorporated into polymer nanostructures either as small molecules dispersed in a polymeric matrix or covalently incorporated into the polymer itself. By incorporating TADF materials into polymers, the solubility, biocompatibility, and oxygen permeability of the material can be readily tuned, and rates of energy transfer within the material can be controlled as well. In this way, the desirable properties of TADF materials can be applied to a wider range of media and combined with other chemical functionality for cellular uptake, targeting, or drug delivery.

In TADF materials, forward and reverse intersystem crossing processes (ISC/RISC) interconvert excitons between singlet and triplet excited states. While transitions between states of unlike spin are generally forbidden, a suitably small singlet-triplet energy gap (ΔE_ST_) allows for state mixing such that these transitions become observable. To design molecules with a small ΔE_ST_, donor (D) and acceptor (A) moieties are arranged so as to limit the overlap between the HOMO and LUMO, minimizing the exchange energy which causes the singlet-triplet energy gap (Endo et al., 2011). TADF is realized when ΔE_ST_ is small enough such that thermal energy alone is sufficient to promote RISC from a triplet state to a higher-energy singlet (Penfold et al., 2018). In applications where a high triplet concentration is detrimental to the material's function, a short emission lifetime and, therefore, high RISC rate (*k*_RISC_) is desirable. El-Sayed's rule for intersystem crossing (ISC) states that transitions between a pure triplet charge transfer state (^3^CT) and pure singlet charge transfer state (^1^CT) are prohibited, and thus even with a small ΔE_ST_, the ^3^CT ^1^CT RISC process is generally slow (Lim et al., 1981). Recent research, however, has found that *k*_RISC_ can be increased by the presence of energetically proximate locally excited triplet (^3^LE) states, such that a small ^3^LE-^3^CT energy gap (|ΔE_3LE−3CT_|) can facilitate conversion between ^3^CT and ^1^CT (Chen et al., 2015; Dias et al., 2016; Gibson et al., 2016; Marian, 2016; Gibson and Penfold, 2017; Hosokai et al., 2017). The converse of this observation is that molecules can also be intentionally designed to have low *k*_RISC_ values, and thus long emissive lifetimes, increasing sensitivity in numerous imaging applications.

The fluorescent mechanism of TADF dyes has significant potential for applications beyond electroluminescent devices, including in sensing and fluorescence imaging. TADF emission lifetimes are typically biexponential, with a short component (<10 ns) due to fluorescence directly from the first excited singlet state (S_1_) as well as a second, delayed component (μs to ms) arising from the RISC process (Dias, 2015; Palmeira and Berberan-Santos, 2018). The involvement of triplet excited states in TADF makes these emitters particularly susceptible to fluorescence quenching from triplet oxygen. This response can be exploited to develop emitters that are sensitive to the O_2_ concentration in a given medium by measuring the change in emission intensity and lifetime of delayed fluorescence (Baleizão et al., 2008; DeRosa et al., 2015). Similarly, *k*_RISC_ depends directly on the temperature of the environment in which the emitter is located, meaning the temperature of a sample can be determined directly from a fluorescence lifetime (Palmeira and Berberan-Santos, 2018). In applications such as these, longer emission lifetimes result in probes with higher sensitivity.

The presence of delayed emission lifetimes without the costly or potentially toxic metallic elements typically found in phosphorescent dyes also makes TADF materials well-suited to time-resolved fluorescence imaging (TRFI) (Zhang et al., 2019). In typical biological systems, autofluorescence from background processes and structures within the cell can significantly reduce the resolution of fluorescent cell imaging (He et al., 2018). These background processes, however, are generally complete in <100 ns, while TADF emission may be detected over a significantly longer timescale. This makes photostable TADF dyes with high brightness highly desirable for TRFI, as background fluorescence can be filtered out by resolving the integrated signal over time or gating the detected luminescence.

In sensing or imaging applications, seclusion of the TADF emitter to control aggregation, limit the influence of environmental factors, and introduce solubility in aqueous systems is the primary goal in host material selection and can be efficiently achieved through incorporation in a polymer host. Characteristics such as charge mobility have minimal effect on sensors unlike small molecule hosts typically used in TADF OLEDs (Chatterjee and Wong, 2019). Such polymer encapsulation can be performed by covalent or non-covalent interactions, with minimal effect on the absorption spectrum of the TADF material. The emission spectrum, however, can be tuned depending on the ability of the polymer matrix to stabilize the emitter's CT excited state. Quantum yield (Φ_F_) values will also vary depending on the polarity of the host polymer and the impact of aggregation on the dye (Gan et al., 2017; Li et al., 2017; Marghad et al., 2019). Indeed, encapsulation within a polymer host results in a reduction of rotational and vibrational degrees of freedom, often resulting in enhanced Φ_F_ and elongated fluorescence lifetimes.

Herein, we discuss recent examples of TADF in polymer nanostructures and thin films. The application of these systems in oxygen and thermal sensing is discussed, as well as methods by which polymer structure can be used to modulate TADF behavior. Progress and current areas of research focus are highlighted, alongside potential avenues for further advancement.

## TADF in Sensors

### TADF Sensors for Oxygen

**Figure 1:**
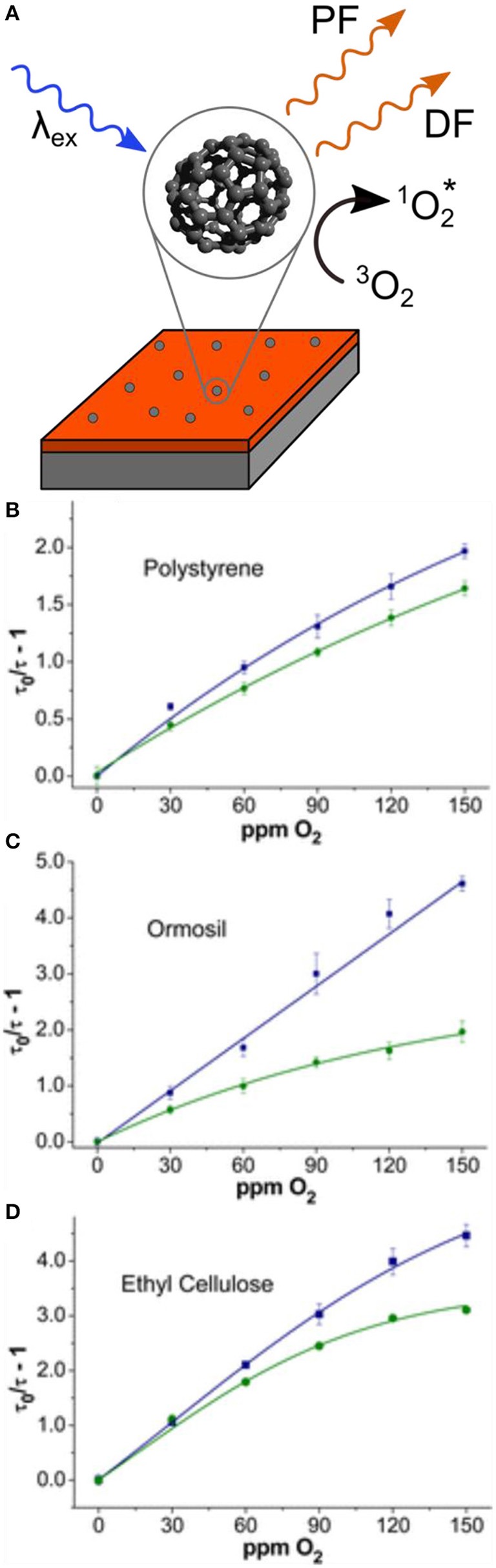
**(A)** Schematic representation of ^13^C_70_ films with excited state quenching by triplet oxygen; **(B–D)** lifetime-based Stern-Volmer plots at 298 K (blue squares) and 333 K (green circles) for ^13^C_70_ in **(B)** PS, **(C)** Ormosil, and **(D)** EC. Adapted from reference (Kochmann et al., 2013) with permission from the American Chemical Society.

Luminescent sensors for oxygen can make use of polymeric hosts to control the interaction of O_2_ with a fluorophore, helping to define the sensitivity and dynamic range of the sensor (Kochmann et al., 2013; Steinegger et al., 2017). TADF was first exploited in oxygen sensing by Wolfbeis and coworkers in 2013, by doping ^13^C_70_ into a series of polymer hosts (Kochmann et al., 2013). ^13^C_70_ has a Φ_F_ of 9%, the highest of any fullerene known, due to increased TADF efficiency from the increased nuclear mass of ^13^C, as well as a nuclear magnetic effect that increases RISC rates (Baleizão and Berberan-Santos, 2011). Moreover, ^13^C_70_ exhibits a high triplet quantum yield of 0.994 and a 170-fold enhancement in Φ_F_ under inert atmosphere (Baleizão and Berberan-Santos, 2011). In this study, ^13^C_70_ was doped into a series of hosts, specifically polystyrene (PS), ethyl cellulose (EC), and organically modified silica (Ormosil), to investigate a range of oxygen permeabilities (P_O2_) from moderate to high. A thermostated flow cell (298 or 333 K) was used to prevent interference from temperature variations, and decay lifetimes in the wavelength range 670–700 nm were monitored as O_2_ concentrations were varied (Figure 1A). In these experiments, Stern-Volmer plots of $\frac{\tau_{0}}{\tau }-1$ vs. O_2_ concentration could be fit using a two-site model which accounted for two distinct probe environments (Figures 1B–D). The linearity of the fit was found to decrease in all films with increasing temperature, due to increased efficiency of RISC and an increased oxygen quenching rate. The lowest limit of detection (LOD) for all films was measured at 0.25 ppm by volume (ppmv) in EC, with upper detection limits of 4,900 ppmv giving the sensors a four order of magnitude dynamic range. These limits of detection were competitive with state-of-the-art sensors using phosphorescent palladium(II) porphyrin complexes in fluorinated polymer hosts, without the use of transition metals.

**Figure 2:**
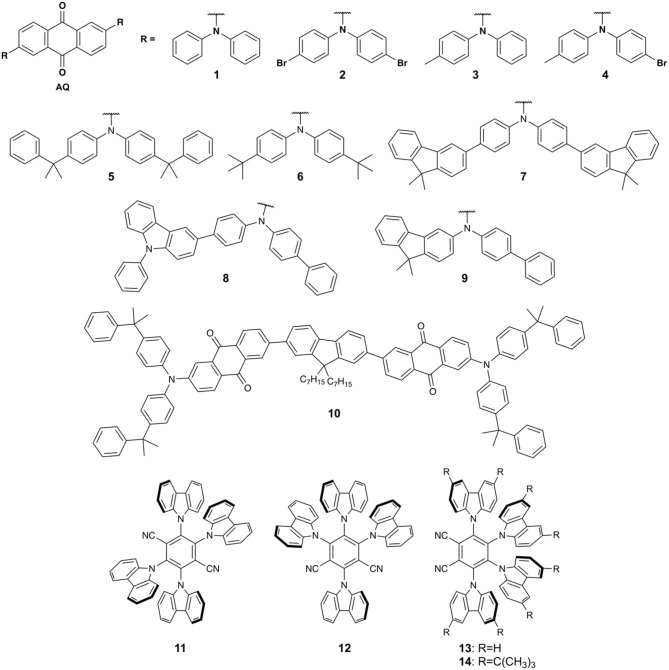
Structures of TADF compounds used by Borisov and coworkers to prepare thin films for oxygen and temperature sensing (Steinegger et al., 2017).

Despite the success of the ^13^C_70_-based films, their low Φ_F_ presents a barrier to more widespread use. An improved sensor design was demonstrated by Borisov and coworkers, who used TADF compounds based on dicyanobenzene (DCB) and anthraquinone (AQ) electron acceptors with arylamine-based electron donors (Figure 2) doped into PS films (Steinegger et al., 2017). The combination of these donors and acceptors affords compounds with absorption in the visible region, high Φ_F_, and good photostability (Zhang et al., 2015). Compounds **1**–**14** dispersed in PS films have π- π^*^ absorption maxima ranging from 329 to 375 nm and charge transfer bands ranging from 440 to 490 nm. Compared to toluene solution the PS host results in blue shifted emission, with DCB dyes **11**–**14** showing emission maxima from 493 to 531 nm and AQ dyes **1**–**10** ranging from 577 to 614 nm in PS films. A significant enhancement in Φ_F_ and excited state lifetimes of **1**–**10** is also observed upon polymer encapsulation due to a restriction in rotational degrees of freedom, decreasing the rate of non-radiative decay. Multicomponent fluorescence lifetimes for films containing **1**–**14** were observed, including a delayed fluorescence component in the μs-ms range. Photodegradation studies revealed that DCB dyes **11**–**14** are highly resistant to photobleaching, while AQ dyes **1**–**10** degrade 2–5 times faster.

To investigate **1**–**14** as potential oxygen sensors, the PS films were tested using a flow cell thermostated at 298 K to eliminate cross-sensitivity to temperature. **11**–**14** have relatively short delayed lifetimes (9–40 μs) and formed devices with low oxygen sensitivity, while **1**–**5** (2,830–5,560 μs lifetimes) gave sensors with sensitivities up to 1.15 hPa^−1^. Similarly to ^13^C_70_-based sensors, a two-site model was used to fit the Stern-Volmer quenching behavior of **1**–**14** in thin films. The Stern-Volmer constant (K_SV_) ranged from 0.00169 to 1.15 hPa^−1^ and results in devices with similar sensitivities to common metal-based phosphorescent oxygen sensors in PS such as platinum(II) pentafluorophenylphophyrin (K_SV_ = 1.81 Pa^−1^) (Borisov and Klimant, 2007), platinum(II) tetraphenyltetrabenzoporphyrins (K_SV_ = 1.65–2.18 Pa^−1^) (Borisov et al., 2008), and ruthenium(II) tris(4,7-diphenyl)-1,10-phenanthroline (K_SV_ = 0. 215 Pa^−1^) (Borisov and Klimant, 2007), however, sensitivities were significantly lower than palladium(II) tetraphenyltetrabenzoporphyrins (K_SV_ = 9.1–10.4 Pa^−1^) (Borisov et al., 2008). Temperature was found to have significant cross-sensitivity with oxygen in these materials however, hindering their application in situations where temperature is uncontrolled.

**Figure 3:**
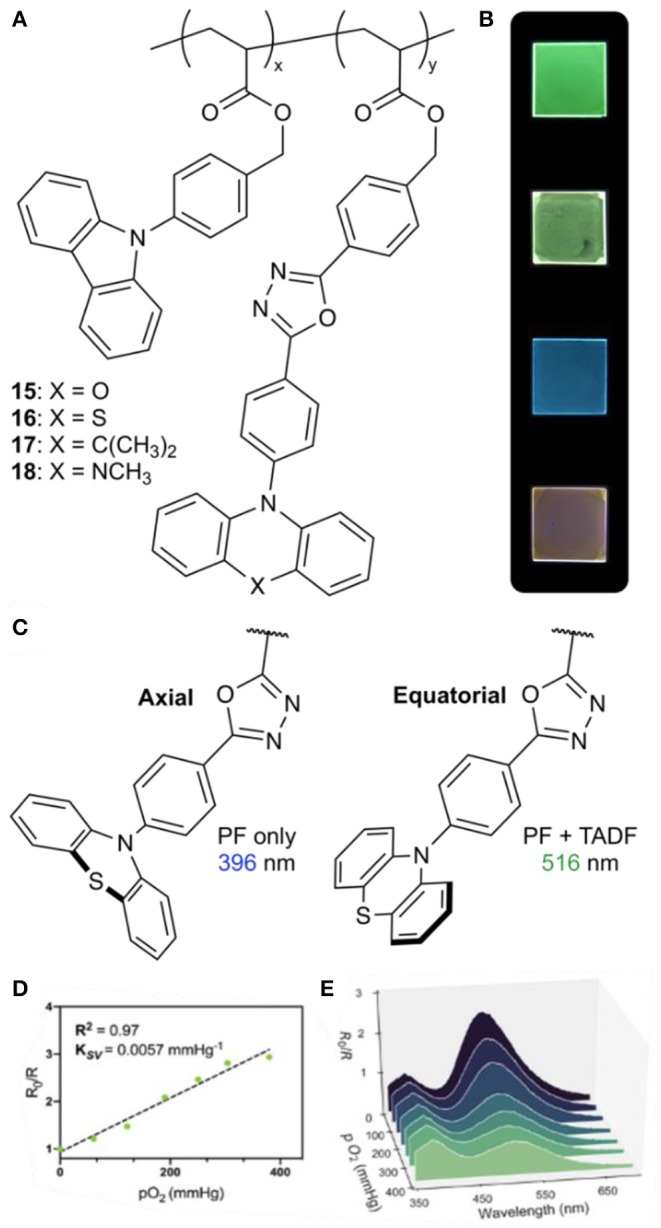
**(A)** Donor-acceptor TADF polymers; **(B)** films of **15**–**18** (from top to bottom); **(C)** illustration of pseudoaxial and pseudoequatorial conformers of phenothiazine and their respective emission wavelengths; **(D)** Stern-Volmer plot comparing the ratio of emission intensity at 516 nm to emission intensity at 396 nm (I_516_/I_396_) at various partial pressures of O_2_ for a film of **16**; **(E)** fluorescence emission response of **16** to O_2_ concentration.

Polymers exhibiting TADF were recently developed in our laboratory that act as ratiometric sensors for oxygen, based on the dual-emissive nature of a TADF chromophore. A series of TADF dopants were prepared using an oxadiazole acceptor paired with either phenoxazine (POZ, **15**), phenothiazine (PTZ, **16**), dimethylacridan (DMAC, **17**), or *N*-methylphenazine (MPAZ, **18**) donors to achieve emission ranging from blue to orange (Figures 3A,B) (Tonge et al., 2020). These materials were doped into a carbazole-based host using copper(0) reversible deactivation radical polymerization (Cu(0)-RDRP), giving molecular weights of ~20 kDa with dispersities from 1.10 to 1.45 (Anastasaki et al., 2016; Sauv et al., 2018; Tonge et al., 2019). Using this method, polymers were synthesized containing 5–15 wt.% TADF emitter, with Φ_F_ values as high as 96%.

The PTZ-containing polymer **16** was found to exhibit dual emission in both solution and the solid state, with emission maxima at 396 and 516 nm. The higher-energy violet emission was found to remain constant regardless of oxygen concentration, while the green emission exhibited oxygen sensitivity characteristic of TADF. There have been several literature reports of PTZ-based donor-acceptor emitters exhibiting dual emission (Daub et al., 2001; Stockmann et al., 2002; Acar et al., 2003; Tanaka et al., 2014; Okazaki et al., 2017; Marghad et al., 2019), arising from two stable conformers of the phenothiazine moiety (Malrieu and Pullman, 1964; Bodea and Silberg, 1968). The pseudoaxial conformer gives a locally excited state with higher-energy emission, while the pseudoequatorial conformer participates in charge transfer, leading to lower-energy emission and TADF (Figure 3C). By taking advantage of the stable emission of the pseudoaxial conformer, oxygen concentration can be determined using the relative intensity of the locally excited and charge transfer emission bands. Stern-Volmer behavior (K_SV_ = 0.0057 mmHg^−1^) was observed using neat thin films of **16** with a linear relationship observed between the emission intensity ratio and O_2_ concentration up to 50% oxygen by volume at 1 atm (Figures 3D,E).

While oxygen-sensitive thin films have potential applications as standalone oxygen sensors, oxygen sensing in aqueous solution would enable applications in biological imaging as well. To demonstrate O_2_-responsiveness in aqueous environments, polymer nanoparticles were prepared by coprecipitating **16** with an amphiphilic polymer, polystyrene-*co*-poly(maleic anhydride) (PS-*co*-PMA). This gave water-soluble “polymer dots” (Pdots) with diameters of 82 ± 38 nm, whose fluorescent properties were similar to those of **16** in thin films. The dual emission of the Pdots showed a responsive color change through the full range of O_2_ solubility in water (up to 44 ppm at 25°C). In contrast to the thin films, this response was non-linear due the distribution of TADF chromophores in the interior of the Pdots and at the surface, giving environments with variable accessibility to O_2_.

### TADF Temperature Probes

**Figure 4:**
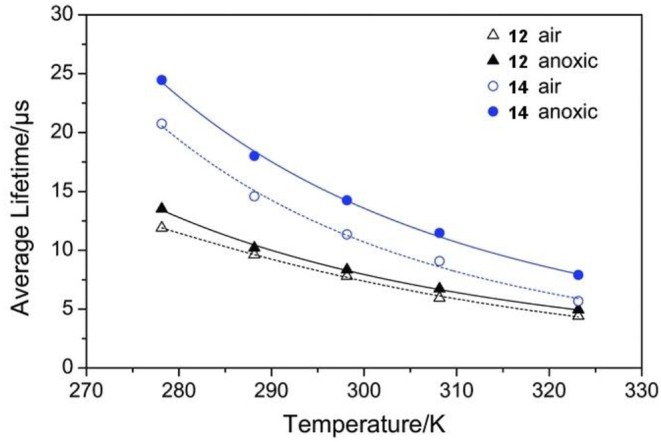
Temperature dependence of the average luminescence decay times of **12** and **14** ERL NPs. Adapted from reference (Steinegger et al., 2017) with permission from Wiley.

In order to employ TADF materials as temperature-sensing probes, the access of oxygen to the dyes must be limited. Borisov and coworkers developed TADF-based temperature probes by encapsulating compounds **1**–**14** in the low-oxygen permeability polymer poly(vinylidene chloride-*co*-acrylonitrile (P(VDC-*co*-AN) (Steinegger et al., 2017). The use of P(VDC-*co*-AN) gives similar absorption maxima compared with PS films, however, a slight red shift in emission is observed due to the higher polarity of the P(VDC-*co*-AN) host. Φ_F_ and lifetimes are reduced compared to PS with the decrease most pronounced in **6**–**9** having Φ_F_ values dropping to ~0.05. Lifetimes as a function of temperature could be fit by an Arrhenius model (Equation 1):

(1) $$\begin{array}{r} \tau ={\left(k_{0}+k_{1}e^{-\frac{\Delta E_{ST}}{k_{B}T}}\right)}^{-1} \end{array}$$

where *k*_0_ is the temperature-independent decay rate, *k*_1_is a pre-exponential factor, *k*_*B*_ is the Boltzman constant, and *T* the temperature in kelvin. The relative sensitivity (% change in τ per K) of the sensors was found to be −1.4 to −4.2 % K^−1^, exceeding the sensitivity of similar europium(III) (Khalil et al., 2004), ruthenium(II) (Liebsch et al., 1999), or chromium(III)-doped (Borisov et al., 2010) yttrium aluminum borate-based thermal sensors (−0.6 to −2.3% K^−1^). **12** and **14** were then incorporated into cationic Eudragit RL100 (ERL) nanoparticles, which are cell-penetrating and provide a route to intracellular fluorescence measurements. ERL is moderately oxygen permeable, and dyes **12** and **14** were chosen to minimize oxygen cross-sensitivity. **12** and **14** ERL dots retained a high sensitivity to temperature, with sensitivities of −2.2 and −2.8 % K^−1^ at 298 K, respectively (Figure 4). A small oxygen cross-sensitivity error of ± 4 K was found for **12** with **14** having a reduced error of ± 1.5 K.

## Fluorescence Lifetime-based Imaging Agents

Delayed emission from compounds free from phosphorescent heavy-metal dopants are of significant interest in biological imaging. The absence of metals decreases the toxicity and reduces the cost of emitters used as imaging probes. In typical biological systems, autofluorescence and other background fluorescence processes cause significant noise that is detrimental to high resolution imaging. These fluorescent processes have lifetimes in the ns range, however, allowing the use of time gating to remove background fluorescence and allowing only emission from species with long excited-state lifetimes to be detected.

**Figure 5:**
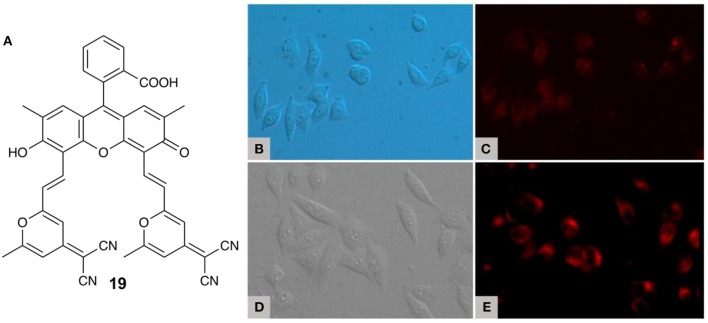
**(A)** Fluorescein derivative **19**; **(B)** bright-field image and **(C)** steady-state luminescence (510–560 nm excitation) of MCF-7 cells immunostained with **19** (20 μM) and BSA (40 μL, 10 mM) at 37°C; **(D)** bright-field image and **(E)** time-resolved luminescence (510–560 nm excitation) of MCF-7 cells stained with **19** (20 μM) and BSA (40 μL, 10 mM) at 37° C. Adapted from reference (Xiong et al., 2014) with permission from the American Chemical Society.

In 2014, Peng and coworkers reported the use of a purely organic fluorophore for TRFI, using fluorescein derivative **19** (Figure 5A) (Xiong et al., 2014). **19** has two absorption features at 485 and 550 nm which density functional theory (DFT) calculations suggest result from transitions between the xanthene and pyran moieties (S_0_ → S_1_) and within the xanthene moiety itself (S_0_ → S_4_). Steady-state emission in aerated acetonitrile gives emission peaks at 525 and 649 nm and a Φ_F_ of 0.28 is observed in ethanol (Xiong et al., 2013). In deoxygenated solution the peak at 649 nm enhances in intensity 24-fold, and with the application of a 100 μs gating time, the 525 nm peak disappears entirely. A multicomponent decay curve is observed with a two-component portion in the μs time scale, with an average lifetime of 22.11 μs. Unsurprisingly **19** shows no DF in aerated 10 mM phosphate-buffered saline (PBS), however, upon the addition of BSA DF is observed. **19** has a suitable size and polarity to enter the hydrophobic cavity of BSA, which secludes the fluorophore from molecular oxygen. Additionally, it has been found that BSA has cell-penetrating qualities that enhance cellular uptake (Xiong et al., 2014). TRFI experiments with nanoparticles (NPs) of **19** encapsulated in BSA using MCF-7 cancer cells demonstrated a large reduction of background fluorescence (Figures 5B–E). Moreover, confocal fluorescence imaging showed extensive permeation of the cell membrane by the TADF NPs, with fluorescence signals localized in the lysosomes. Colocalization experiments using a commercial lysosome tracking dye (LysoSensor Green DND-189) also showed good signal overlap from the two emitters. Finally, biological toxicity of free **19** using the MCF-7 cell line showed minimal cell death observed after 10 h, making this compound promising for applications in TADF-based TRFI.

**Figure 6:**
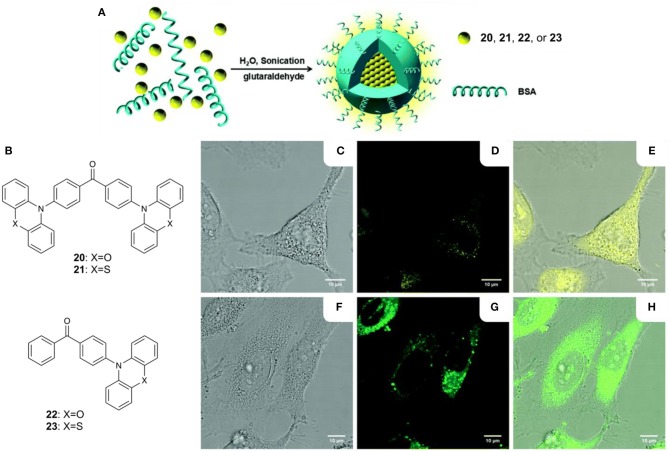
**(A)** Fabrication of BSA NPs; **(B)** AIE TADF compounds **20**–**23**; confocal fluorescence images of HeLa cells after incubation with **(C–E)** BSA NPs of **21** (10 μM of **21**) and **(F–H)** BSA NPs of **22** (10 μM of **22**) at 37°C for 0.5 h; **(C,F)** bright-field images; **(D,G)** fluorescence images; **(E,H)** merged bright field and fluorescence images; scale bar = 10 μm. Adapted from reference (Gan et al., 2017) with permission from The Royal Society of Chemistry.

While the use of BSA as a biopolymer encapsulation matrix enables imaging in aqueous environments, a low Φ_F_ is often observed from the resulting NPs. Tang and coworkers argued that aggregation-caused quenching (ACQ) resulting from high concentrations of fluorophore inside the BSA NPs was responsible, and proposed that this problem could be resolved using TADF molecules that exhibit aggregation-induced emission (AIE) (Gan et al., 2017). Compounds with a benzophenone acceptor and POZ or PTZ donors were investigated, giving materials **20–23** with highly twisted conformations which inhibit π stacking (Figures 6A,B). Solid-state emission maxima for these compounds ranged from 544 to 558 nm, with Φ_F_ values from 0.14 to 0.24 and delayed lifetimes between 0.66 and 1.36 μs. Encapsulation in BSA gave TADF NPs in water with diameters ranging from 109 to 150 nm. Confocal laser scanning microscopy (CLSM) showed that the TADF NPs effectively stain HeLa cells and show green to yellow emission within the cytoplasm (Figures 6C–H). Moreover, differences in intracellular viscosities result in the measurement of two distinctive lifetime regions (~1,000 and ~2,000 ps, respectively) by TRFI.

While the use of AIE materials facilitates imaging at high brightness within BSA nanoparticles, the cross-sensitivity with intracellular viscosity suggests that the TADF dyes are not well-isolated from the cytoplasm. Alternatively, the use of Pdots formed from amphiphilic polymers offers the ability to encapsulate organic molecules within a hydrophobic core, surrounded by a hydrophilic, biocompatible corona (Kuo et al., 2015; Massey et al., 2015; Yu et al., 2017). The amphiphilic polymer acts as a barrier against oxygen and polar molecules, allowing for time-gated imaging under biological conditions. Moreover, the exclusion of TADF dyes from the cytoplasm prevents cell damage from singlet oxygen produced through triplet quenching or innate toxicity from the fluorophores themselves.

**Figure 7:**
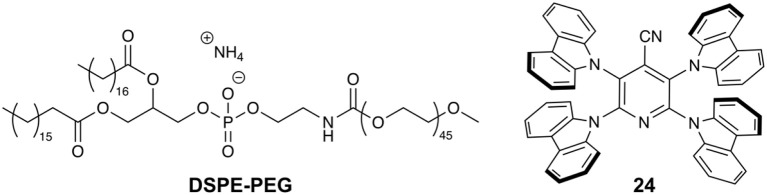
The amphiphilic polymer DSPE-PEG and TADF **24** used by Huang and coworkers (Li et al., 2017).

Huang and coworkers investigated the use of TADF Pdots for biological imaging by employing the amphiphilic polymer 1,2-distearoyl-*sn*-glycero-3-phosphoethanolamine-*N*-[methoxy(polyethylene glycol)-2000] (DSPE-PEG, Figure 7) and the TADF material 2,3,5,6-tetracarbazole-4-cyano-pyridine (**24**) (Li et al., 2017). **24** has been shown to have increased emission in the solid state (Φ_F_ = 54.9%) compared with chloroform solution (Φ_F_ = 24.7%) and, therefore, would not be negatively affected by aggregation within the Pdot. **24** displays green emission, absorbs in the visible range, and exhibits a DF lifetime of 8.3 μs in deoxygenated toluene. DSPE-PEG and **24** were precipitated to form Pdots which had a spherical morphology with an average diameter of 17 nm by TEM and 23 ± 5 by DLS. Pdots of **24** show a CT absorption band at ~450 nm in water which is red-shifted compared to free **24** in THF, and emission at 535 nm with Φ_F_ = 0.38. In aerated water a biexponential decay is observed with a long lifetime component of 9.4 μs. Photobleaching studies revealed Pdots of **24** retained 90% of their original fluorescence intensity after 45 min of laser irradiation (55.6 mW cm^−2^ at 405 nm).

Pdots of **24** show good Φ_F_, long lifetimes, and resistance to photobleaching, however, their stability and biocompatibility had to be investigated. To test the chemical stability of the Pdots fluorescence measurements were made in Milli-Q water, PBS, and tris-acetate buffer. Dulbeco's modified Eagle medium (DMEM) was also used to simulate physiological conditions. Compared to the Milli-Q water solution, the fluorescence intensity was minimally affected by PBS and tris-acetate buffer and only decreased by 20% in DMEM. Moreover, in all cases the fluorescence intensity remained stable after 48 h in solution at 37°C. Toxicity studies were also conducted by incubating HeLa cells with variable concentrations of Pdots for 24 h. A maximum concentration of 9 μM could be used while retaining >80% cell viability and concentrations of 4.5 μM or lower resulted in minimal cell death.

**Figure 8:**
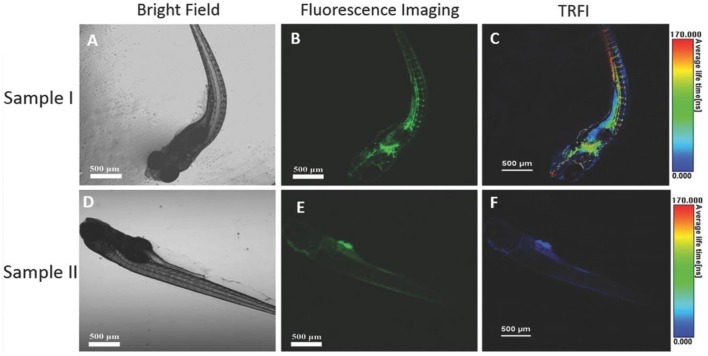
Confocal fluorescence images of zebrafish: **(A–C)** zebrafish injected with **24** Pdots; **(D–F)** zebrafish without any Pdots injected; **(A,D)** bright field images; **(B,E)** confocal fluorescence images recorded with 480–580 nm bandpass filters for **24** Pdots upon excitation at 405 nm; **(C,F)** fluorescence lifetime images. Reproduced from reference (Li et al., 2017) under the Creative Commons CC-BY license.

The effectiveness of **24** as an imaging probe for *in vitro* studies was determined using HeLa cells as an example system. The cells were incubated with Pdots of **24** (0.5 μM) in PBS for 2 h. Short-lived background fluorescence is observed from the cytoplasm while emission from **24** is detected from the cell membrane with an average lifetime of 165 ns. Co-staining with **24** Pdots and a cell membrane marker, 1,1′-dioctadecyl-3,3,3′,3′-tetramethylindocarbocyanine perchlorate (DiIC18), gives overlapping emission confirming the localization of the Pdots. It was also proposed that the lipophilicity of DSPE-PEG causes Pdots near the cell to interact with the cell membrane. During this process, dye **24** can transfer from the Pdot into the cell membrane, where it is retained. This mechanism is consistent with the observation that excited state lifetimes shortened significantly during imaging compared to free Pdots of **24** measured in water. Finally, the membrane-labeling property of Pdots of **24** was explored by confocal imaging and TRFI of a living zebrafish, giving a clear picture of the vascular network of the animal with minimal background fluorescence (Figure 8).

**Figure 9:**
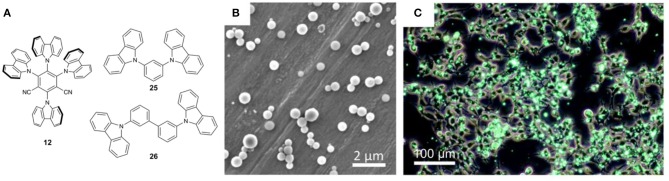
**(A)** TADF emitter and host materials; **(B)** SEM image of 6 wt.% glassy Pdots (2 μm scale bar); **(C)** combined phase contrast image of glassy Pdots in HEK293 cells after 12 h of incubation (100 μm scale bar). Adapted from reference (Tsuchiya et al., 2019) with permission from The Royal Society of Chemistry.

While polymer encapsulation often reduces Φ_F_ values and red-shifts the emission of TADF dyes, Adachi and coworkers recently applied concepts from OLED design to address these issues and improve the performance of TADF Pdots (Tsuchiya et al., 2019). Organic semiconductor host materials are commonly used in OLEDs to isolate emitters and prevent aggregation quenching effects, and it was hypothesized that these same ideas could be used to increase Φ_F_, reduce aggregation, and enhancing the stability of the TADF dyes in Pdots. By incorporating TADF dyes into the glassy semiconductor host **25** within DSPE-PEG, TADF dye **12** (Figure 9A) could be effectively protected from the surrounding medium and aggregation could be prevented. The combination of **12**+**25** had previously been shown to be an effective dye/matrix combination in the emissive layer of OLEDs (Uoyama et al., 2012; Nakanotani et al., 2013), suggesting that energy transfer from **25** to **12** should be highly favorable. Aqueous mixtures of **12**, **25**, and DSPE-PEG were heated to 180°C under pressure and then rapidly cooled to melt and subsequently solidify **12** and **25** within the Pdot. This produced spherical particles (Figure 9B) with an average diameter of 371 nm by DLS. The use of host **26** in place of **25** was also attempted, but it was found that the high melting point of **26** (268.7°C) and lower crystallization temperature (131.3°C) resulted in microcrystal formation during processing. Aqueous glassy Pdots of **12+25** gave slightly red-shifted emission and higher Φ_F_ (λ_em_ = 516 nm and Φ_F_ = 0.94) compared with **12** alone in toluene solution (λ_em_ = 498 nm and Φ_F_ = 0.83), suggesting that the strategy was promising. Importantly, the fabrication of glassy Pdots in argon-saturated water was found to be essential as the use of aerated water during fabrication resulted in a drastic decrease in Φ_F_ of 32%. A delayed lifetime of 3.1 μs was observed from the glassy Pdots which was nearly insensitive to the presence of oxygen. High photo-stability was observed with intensity dropping by only 25% after 115 min of irradiation. Minimal cytotoxicity was observed with HEK293 cells, consistent with the work of Huang and coworkers (Li et al., 2017) and the known biocompatibility of DSPE-PEG. The Pdots showed strong fluorescence in HEK293 cells (Figure 9C) with cells retaining their emission after 7 days of culturing. Long-term traceability experiments were also conducted with HEK293 cells to utilize the high stability and low cytotoxicity of the glassy Pdots. The glassy Pdots were observable for 21 days with cell viability matching that of the control cells. Interestingly, cell membrane labeling was not observed in this study, despite the use of the same amphiphilic polymer employed in the earlier study by Huang and coworkers (Li et al., 2017).

**Figure 10:**
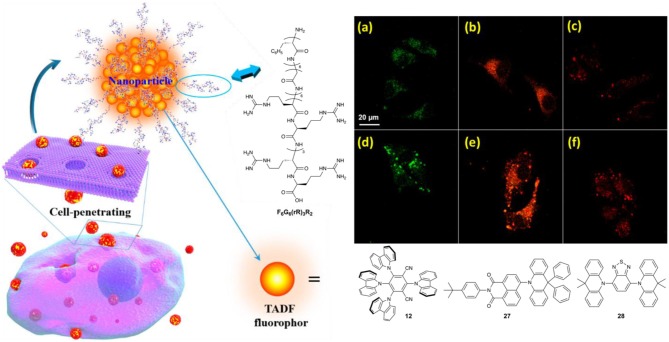
Schematic of CPP Pdots fabricated from F_6_G_6_(rR)_3_R_2_ and TADF emitters **12**, **27**, and **28**. CLSM images of HeLa cells after incubation with CPP encapsulated **(a,d) 12**, **(b,e) 27**, **(c,f)**, and **28** Pdots for **(a–c)** 5 min and **(d–f)** 15 min. [**12**] = [**27**] = [**28**] = 2 μg mL^−1^. Adapted from reference (Zhu et al., 2018) with permission from the American Chemical Society.

Despite the utility of non-glassy and glassy Pdots in TRFI imaging, both require several hours of incubation time to effectively stain cells due to their low membrane permeability. Zhao and coworkers used a cell-penetrating peptide (CPP) as an alternative encapsulating amphiphile to address this issue (Zhu et al., 2018). The CPP (F_6_G_6_(rR)_3_R_2_) consisted of a hydrophilic octamer [(rR)_3_R_2_] formed from L-arginine (R) and D-arginine (r), a hexamer of glycine (G), and a hydrophobic hexamer of phenylalanine (F) (Figure 10) (Zhu et al., 2018). (rR)_3_R_2_ was used as the hydrophilic moiety of the CPP amphiphile due to previous research showing the high biocompatibility of the fragment (Ma et al., 2012). The TADF dyes **12**, **27**, and **28** were used as they have been successfully incorporated into high performance OLEDs and give a range of emission wavelengths. Aqueous CPP Pdot solutions were formed using a reprecipitation method giving particles 92 to 177 nm in diameter by DLS. **12**, **27**, and **28** Pdots showed emission at 555, 607, and 657 nm with Φ_F_ values of 0.12, 0.025, and 0.008, respectively. Emission is red-shifted and Φ_F_ values were drastically reduced compared to their values in toluene solution due to the effects of aggregation and the polar amide backbone of F_6_G_6_(rR)_3_R_2_. All fluorescence decays were multi-exponential with delayed lifetimes ranging from 1.8 to 93.7 μs. No significant change in lifetime was observed between aerated and deoxygenated solution.

HeLa and 3T3 cells were used to test the cell-penetrating TADF PDots as imaging agents. As expected, low cytotoxicity was observed from all three TADF Pdots using both cell lines with 24 h of incubation at 37°C. Cell viabilities remained above 80% with up to 10 μg/mL of TADF dye. After only 5 min of incubation with these Pdots (2 μg/mL of TADF dye) HeLa cells showed fluorescence by CLSM, with a 44% or higher fluorescence-positive rate by flow cytometry (Figures 10A–C). With an incubation time of 15 min the fluorescence-positive-rate rose to a minimum of 54%, cellular accumulation of the TADF Pdots increased dramatically (Figures 10D–F). Cellular uptake was also conducted with 3T3 cells with similar results. In this study, TRFI could be used with a delay time of only 50 ns to remove background fluorescence, yet delays as long as 500 ns could also be used with observable emission.

### Controlling TADF With Polymer Morphology

Given the applications of TADF materials in polymers for imaging and sensing, our group has recently explored methods for modulating TADF behavior in polymers by controlling polymer morphology (Tonge and Hudson, 2019). With typical TADF emitters it can be challenging to achieve turn-on/off delayed fluorescence emission using a small molecule emitter in which the donor and acceptor are bound covalently. In 2017, Wang and coworkers reported that TADF behavior could be observed in non-conjugated copolymers of discrete donor and acceptor monomers using a through-space charge transfer (TSCT) mechanism (Shao et al., 2017). Wang and coworkers hypothesized that minimization of donor-acceptor overlap and a small ΔE_ST_ could be achieved simply by copolymerizing electron-rich and electron-poor organic semiconductors, using a polymer backbone to achieve close D-A contact. This strategy was used successfully to fabricate high-efficiency blue OLEDs, as well as achieving color-tunable emission by modifying the acceptor electronic properties (Hu et al., 2019). Excited by this new paradigm in TADF polymer research, we sought to determine if alternative polymer morphologies could be used to control TSCT emission, using bottlebrush copolymers (BBCPs). Consisting of polymeric side chains attached to a linear backbone, the steric interactions of the densely packed side chains induce an extended backbone conformation, allowing for discrete nanoscale domains to be prepared along the length of the polymer.

**Figure 11:**
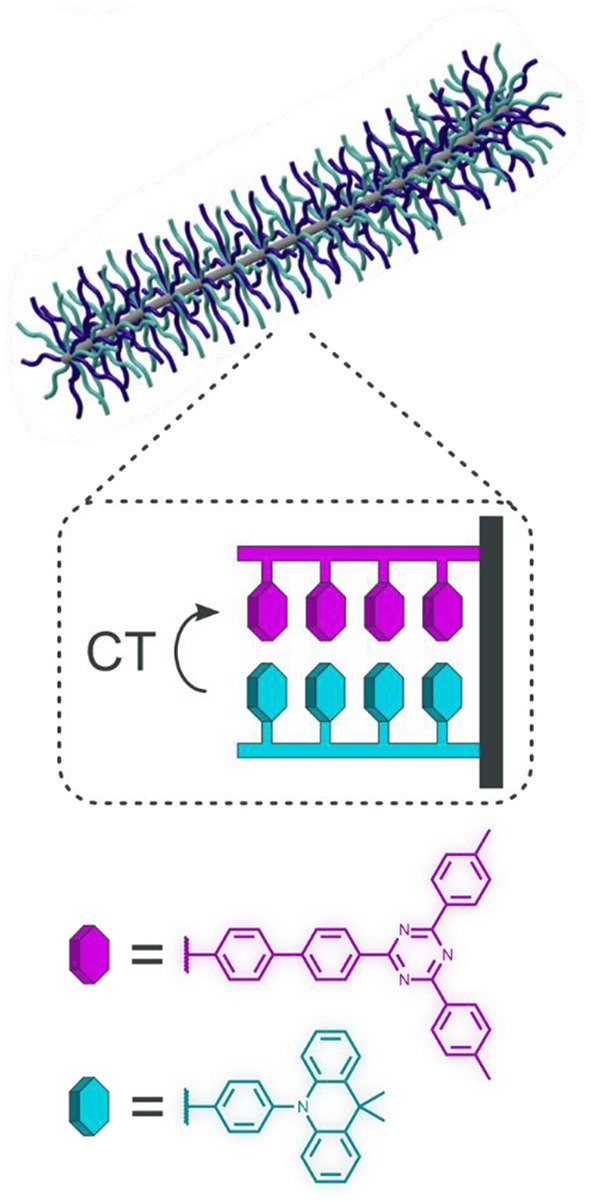
Schematic of TSCT within BBCPs.

Using this approach, we designed a series of BBCPs composed of acridine-based donors and triazine-based acceptors, varying the degree of donor-acceptor blending along the BBCP to control TSCT (Figure 11) (Tonge and Hudson, 2019). Grafting density along the bottlebrush backbone was maximized by employing a grafting-through polymerization strategy, in which short linear polymers functionalized with reactive norbornene end groups (termed “macromonomers”) are prepared first, then polymerized using ring-opening metathesis polymerization to give the BBCPs. Three bottlebrush copolymers containing 50% acridine-based donor and 50% triazine-based acceptor were prepared: one bottlebrush in which the macromonomers themselves were random D-A copolymers, another mixed arm (or “miktoarm”) bottlebrush consisting of a random copolymer of D or A macromonomers, and a third in which the D and A macromonomers were polymerized to give a diblock BBCP.

These blended BBCPs were found to exhibit unique photophysical properties as a direct result of their morphology. The diblock copolymer displayed only prompt fluorescence in both the solution and solid state, while the miktoarm and random copolymers showed some degree of TSCT emission. The fully blended BBCP exhibited only TSCT emission in both the solid state and in solution, as near-complete mixing was ensured by the microstructure of the bottlebrush. Interestingly, by segregating the donor and acceptor functionalities in the diblock polymer, minimal interpenetration of these blocks was observed in the solid state or in solution, resulting in emission from only the monomers themselves. This result is noteworthy as a simple spin-cast film of a blend of linear donor and acceptor polymers gives both monomer and TSCT emission. This implies that using a bottlebrush backbone, the degree of interaction between these monomers can be controlled simply by modulating polymer morphology.

**Figure 12:**
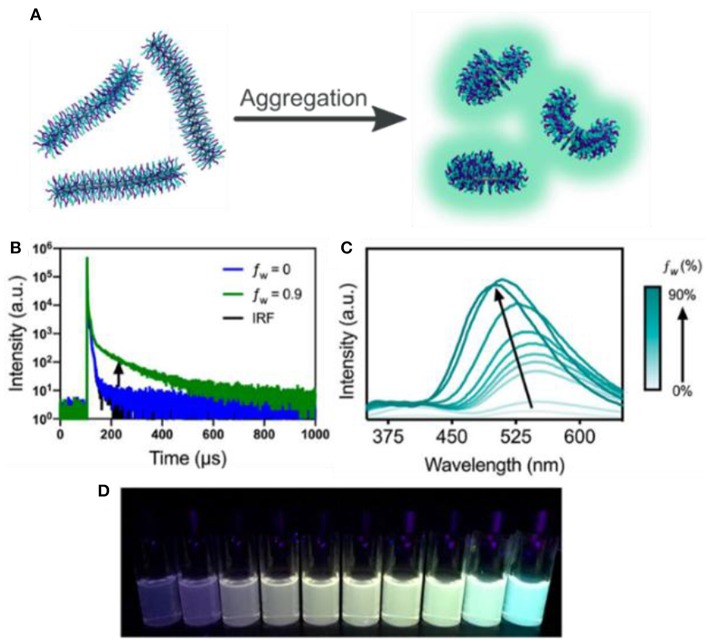
**(A)** Schematic of conformational changes in the aggregated state; **(B)** lifetime increase from increasing water fraction (*f*_w_ = 0 to 90%) at a concentration of 0.02 mg mL^−1^; **(C)** aggregation induced emission of miktoarm bottlebrush copolymer (0.02 mg mL^−1^), normalized such that the maximum intensity at *f*_w_ = 0% is set to 1 and all other spectra are scaled accordingly; **(D)** image of the fluorescent emission of the corresponding aggregated samples from *f*_w_ = 0–90%. λ_ex_ = 313 nm.

The TSCT in these bottlebrushes was also found to exhibit a substantial AIE effect, particularly in the case of the miktoarm copolymer (Figure 12A). When dissolved in THF, the miktoarm polymer demonstrates an approximately equal mix of prompt emission and TSCT emission, as adjacent chains are spread out due to favorable interactions with the solvent. Upon aggregation in the presence of water, however, the solvent is excluded, leading to a significant increase in TSCT emission and a corresponding decrease in prompt fluorescence (Figures 12B–D). This results in a 15.8-fold increase in brightness for the delayed fluorescence peak, which is not observed in the case of the diblock bottlebrush where TSCT is prevented by the polymer morphology. The fluorescence lifetime was also found to increase substantially upon aggregation of the random and miktoarm BBCPs, a feature that can potentially be taken advantage of for fluorescence lifetime imaging and other sensing applications.

## Conclusions and Outlook

Alongside their applications in OLEDs, TADF materials are becoming increasingly attractive as emitters for sensors and imaging probes. Embedding TADF materials in polymer films or nanostructures has unlocked many such applications, allowing these materials to be compatible for applications such as biological imaging, and presenting new avenues for controlling the photophysics of the TADF dopant. Taking advantage of triplet quenching processes, polymer thin films have been demonstrated as effective sensors for molecular oxygen with tunable sensitivity depending on the polymer host chosen. Using a TADF material with dual emission, it has been demonstrated that oxygen sensing is also possible without internal calibrants, enabling color-contrast imaging with water-soluble nanoparticles. Using polymers with low oxygen permeability, effective temperature sensors with high sensitivity have also been developed with dynamic ranges tunable simply by the TADF dopant used. The effect of TADF dopant concentration on oxygen permeability and TADF is understudied and would bring benefit to the community.

Using BSA to encapsulate TADF emitters, water-soluble, biocompatible nanoparticles can be formed which permit the use of TADF compounds in TRFI. By using TADF compounds exhibiting AIE behavior or by incorporating glassy semiconductor hosts into the nanoparticle, the detrimental effects of aggregation can be alleviated and imaging using TADF nanoparticles can be improved. TADF Pdots can be formed from amphiphilic polymers to retain the high brightness of the dyes in aqueous media, and cell-penetrating peptides can be used as effective encapsulating agents to increase cellular uptake. Finally, polymer morphology alone has recently been found to be an effective method for controlling TSCT TADF, either by aggregation of TADF-based bottlebrush fibers, or by controlling the extent of D-A mixing along a bottlebrush backbone to give the level of TADF desired.

Stimuli-responsive polymers exploiting TADF have now opened up a wide range of future avenues for research in sensors and biological imaging. Time-gated detection is now possible in aqueous environments at high brightness without the use of phosphorescent metal complexes, and in some cases without the need for colocalization dyes for calibration. This is sure to create opportunities for TADF materials in the growing field of theranostics, wherein chemistries for cell targeting, imaging, and therapy are combined onto a single nanoparticle carrier. In imaging applications, the potential for TADF dopant leakage has yet to be addressed, with current implementations relying on the poor solubility of TADF dyes in water to prevent emitter loss from the polymer particle. Covalent incorporation of TADF dyes into the polymer structure would prevent this entirely, and presents a promising strategy deserving of further research. Moreover, imaging probes with high stability to photobleaching, high Φ_F_, and enhanced cellular uptake have yet to be achieved and are sure to be actively sought by the community. Overall, we believe that stimuli-responsive polymers exhibiting TADF have only begun to be explored, and are sure to have a bright future in biological, analytical, and photophysical research.

## Author Contributions

This review was written by NP, CT, and ZH.

### Conflict of Interest

The authors declare that the research was conducted in the absence of any commercial or financial relationships that could be construed as a potential conflict of interest.
